# Exopolysaccharides from *Pseudomonas tolaasii* inhibit the growth of *Pleurotus ostreatus* mycelia

**DOI:** 10.1515/biol-2022-0601

**Published:** 2023-05-23

**Authors:** Yanyan Xu, Taimei Yao, Haiyan Yan, Longzuo Xin

**Affiliations:** College of Agriculture and Forestry Science and Technology, Hebei North University, Zhangjiakou 075000, China

**Keywords:** *Pseudomonas tolaasii*, exopolysaccharides, *Pleurotus ostreatus*

## Abstract

In the present study, the effect of exopolysaccharides (EPSs) extracted from *Pseudomonas tolaasii* on the growth of *Pleurotus ostreatus* mycelia was determined. *P. ostreatus* mycelia was cultivated with different concentrations of *P. tolaasii* EPSs, and their mycelial growth rate, protein content, and enzyme activity were measured and compared. The results showed that EPSs inhibited the growth of *P. ostreatus*. The proline and vitamin C contents of *P. ostreatus* increased at an EPS concentration of 40%. The cellulase, α-amylase, protein, and glucose utilisation rates of *P. ostreatus* gradually decreased with the increase in EPS concentration. Altogether, *P. tolaasii* EPSs had a significant inhibitory effect on mycelial growth. Therefore, we concluded that in addition to tolaasin, EPSs may also be the virulence factors responsible for the pathogenesis of *P. tolaasii*.

## 1 Introduction

*Pseudomonas tolaasii* is the causative agent of brown blotch disease (BBD) in *Pleurotus ostreatus*, *Agaricus bisporus*, *Flammulina velutipes*, and *Pleurotus eryngii* [1]. With the advancements in production methods and large-scale cultivation, the incidence of bacterial BBD continues to increase. BBD is known to affect the yield and quality of edible fungi [2].

BBD can occur at any time during the growth of *P. ostreatus* fruiting bodies. In *P. ostreatus*, BBD is caused by the secretion of the extracellular lipodepsipeptide toxin tolaasin by *P. tolaasii* [3,4] through a non-ribosomal mechanism that binds multiple enzymes [5–9]. In addition, tolaasin synthesis is controlled by gene regulation [10,11]. Extracellular toxins cause yellow-brown spots on the surface of mushrooms, including *P. ostreatus*, by changing their permeability or directly destroying the cell membrane to form cavities, which affects the transmembrane transport of ions [12,13]. These yellow-brown spots are caused by the infected fruit body in the process of fruit body damage or senescence, when the phenolic substances in the fruit body are oxidised into quinones and aggregated into melanin. Tolaasin is thought to be the main pathogenic cause of BBD in *P. ostreatus* [12].

Some extracellular factors also affect *P. ostreatus* mycelia, assisting the invasion of *P. ostreatus* mycelium by *P. tolaasii*. Aminobenzene [14], Tolaasin II [15], methanethiol, dimethyl disulphide, and 1-undecene [16] are factors potentially involved in the development of BBD symptoms.

The aim of the present study was to provide a theoretical basis for further studies on the pathogenic mechanism of *P. tolaasii* as well as to lay a foundation for the prevention and control of BBD in *P. ostreatus*. Exopolysaccharides (EPSs) were extracted from the extracellular fluid of *P. tolaasii*. *P. ostreatus* mycelia were cultivated with different EPS concentrations in a solution to determine their growth rate; intracellular proline and vitamin contents; amounts of extracellular carboxymethyl cellulose (CMC) enzymes, α-amylase, and laccase; glucose utilisation rate; and protein content. Various virulence factors were used to elucidate the pathogenic mechanisms of *P. tolaasii*.

## 2 Materials and methods

### 2.1 Bacterial strains and culture conditions

A single colony of *P. tolaasii* (CGMCC 1.19361) was inoculated in the Luria–Bertani (LB) liquid medium and incubated at 30°C with shaking it for 48 h.

### 2.2 Extraction of *P. tolaasii* EPSs

The cultivated bacterial liquid was placed in a 100°C water bath for 10 min to inactivate the enzymes. The bacterial solution was centrifuged for 15 min at 4°C and 8,000 rpm. The supernatant was collected after centrifugation, 12% trichloroacetic acid solution of half the volume of the supernatant was added, and the proteins were removed from the supernatant after stirring for 30 min. Following centrifugation at 4°C at 8,000 rpm for 15 min (Sigma 3-18KS, Harzberg am Harz, Germany), the supernatant was collected, mixed with 2–3 times its volumes of 95% ethanol solution, and precipitated overnight. Then, the solution was centrifuged at 4°C and 8,000 rpm for 15 min to obtain a precipitate containing EPSs, after which it was weighed [11,17].

Based on the quality of the EPSs, the precipitate was diluted with sterile water to prepare EPS solutions with different concentrations. After preparation, the bacteria were removed using a 0.22 µm filter (Millipore, MA, USA) and used as a backup (Table 1).

**Table 1:** Preparation of different EPS concentrations

Concentration (g/mL)	EPS (g)	Sterile water (mL)
3%	0.3	10
5%	0.5	10
10%	2	20
20%	2	10
30%	3	10
40%	4	10

### 2.3 Growth rate of mycelia with EPSs

*P. ostreatus* was inoculated on a potato dextrose agar (PDA) plate and incubated at 25°C until the plate was covered. Then, 100 μL of the solutions with different EPS concentrations (0, 3, 5, 10, 20, 30, or 40%) was added to the PDA plate. Sterile water was used as the control. Plate-grown *P. ostreatus* was perforated with a perforator (1 cm diameter), inoculated on the PDA with an inoculum tweezer (three replicates per concentration), and incubated at 25°C. Mycelial growth was measured by the cross–cross method and averaged from day 2 [18]. Mycelial growth was determined using the following formula: Growth rate of mycelium = Colony diameter (mm)/day.

### 2.4 Intracellular crude extraction from *P. ostreatus* mycelia

*P. ostreatus* was inoculated into the PDA media containing different EPS concentrations and incubated at 25°C. The mycelia were collected at 10 dpi, grinded with liquid nitrogen and mixed with ten times volume of distilled water, and then centrifuged at 4°C and 4,000 rpm for 10 min. The supernatant was collected as a crude extraction solution.

### 2.5 Measurement of proline and vitamin C content in *P. ostreatus* mycelia

A standard proline solution (100 μg/mL) was prepared and diluted to the desired concentration. Optical density (OD) was measured at a wavelength of 520 nm (Liuyi, Beijing, China) using the ninhydrin colorimetric method. Using proline content as the abscissa, the density value was plotted on the ordinate as a standard curve. The proline content was determined according to method 979.20 of the Association of Official Analytical Chemists [1]. The results are expressed as μg proline/1,000 g mycelium. The vitamin C content of the mycelia was determined using a spectrophotometric procedure (Liuyi), as described by Bajaj and Kaur [19].

### 2.6 Extraction of extracellular crude enzymes of *P. ostreatus* mycelia

The *P. ostreatus* strain cultured on the PDA plate was peeled off and placed in prepared liquid tubes at different EPS concentrations (0, 10, 30, and 40%). The culture medium was shaken at 25°C and the liquid medium was prepared as shown in Table 2. After culturing for 3 days, the liquid medium was centrifuged at 4°C and 4,000 rpm for 15 min, and the supernatant was collected as an extracellular crude enzyme solution.

**Table 2:** Preparation of different EPS concentrations in liquid medium

Treatment (%)	EPS (g)	Sterile water (mL)	PD (mL)
10	1	9	1
30	3	9	1
40	4	9	1

### 2.7 Extracellular enzyme activity of *P. ostreatus* mycelia

#### 2.7.1 Carboxymethyl cellulase (CMCase) activity

The CMCase activity of the mycelia was determined using CMC (Sigma-Aldrich Corporation, St Louis, MO, USA) [20]. CMCase (EC 3.2.1.4) hydrolyses the β-1.4 glucosidic linkage in the carboxyl cellulose molecule and releases the reducing sugar (glucose), which reacts with 3,5-dinitrosalicylic acid (DNS) [21] and produces a colour change proportional to the amount of released reducing sugar (glucose), which is proportional to the enzyme activity in the sample. The amount of reducing sugar produced was determined by measuring the absorbance at 540 nm (using glucose as a standard) (Liuyi) to measure the extent of enzyme activity.

#### 2.7.2 α-Amylase activity

The α-amylase activity of the extracellular crude enzyme solution was measured using the 3,5-DNS method [22]. The OD of the samples was measured at a wavelength of 590 nm (Liuyi) [23]. The results are expressed as U/L.

#### 2.7.3 Laccase activity

Laccase activity of the extracellular crude enzyme solution was determined using 2,6-dimethoxyphenol as a substrate, as described previously [10]. The OD was recorded at a wavelength of 468 nm (Liuyi) [24]. The amount of enzyme required to oxidise 1 μmol of substrate per minute was defined as 1 U enzyme activity.

### 2.8 Extracellular protein and glucose utilisation rate of *P. ostreatus* mycelia

After centrifugation, the crude enzyme solution was assayed for protein content using the Coomassie Brilliant Blue Kit A054-2 in accordance with the manufacturer’s instructions (Nanjing Jiancheng Bioengineering Institute, Nanjing, China). The extracellular sugar content was determined using a biosensor (Institute of Biology, Shandong Academy of Sciences, Jinan, China). After calibration of the standard product, the concentration of a 25 μL sample aliquot was determined and recorded.

### 2.9 Data analysis

All experiments were performed in triplicates. The data were analysed using analysis of variance and multiple comparison tests using Excel (Microsoft Corp., Redmond, WA, USA) and the SPSS software (PSS, Inc., Chicago, IL, USA). The confidence level was set at 95% (*p* ≤ 0.05). The results are presented as mean ± standard deviation (SD).

## 3 Results

### 3.1 Effect of *P. tolaasii* EPSs on the growth rate of *P. ostreatus*

**Figure 1:**
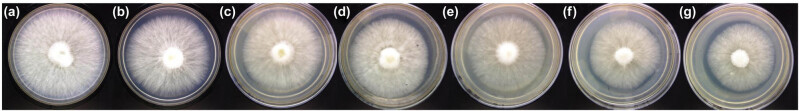
Growth of *P. ostreatus* mycelium at different EPS concentrations: (a) control, (b) 3% EPS solution, (c) 5% EPS solution, (d) 10% EPS solution, (e) 20% EPS solution, (f) 30% EPS solution, and (g) 40% EPS solution.

The growth rates differed significantly under different EPS concentrations (Table 3). The hyphae growth rate of *P. ostreatus* decreased with the increase of EPS concentration. The negative control of *P. ostreatus* mycelia had a maximum growth rate of 18 mm/day, whereas the lowest growth rate of 14.2 mm/day was recorded under the 40% EPS concentration treatment. On the plate with EPS, the hyphae were sparse and the colour turned yellow (Figure 1). Based on these findings, we concluded that the EPS of *P. tolaasii* significantly inhibited the growth of *P. ostreatus* hyphae.

**Table 3:** Growth rate of *P. ostreatus* mycelium at different EPS concentrations

EPS concentration (g/mL)	Growth rate (mm ± SD/day)
0%	18.0 ± 0.000^a^
3%	16.5 ± 0.212^b^
5%	16.2 ± 0.261b^c^
10%	15.9 ± 0.216^c^
20%	15.0 ± 0.151^d^
30%	14.3 ± 0.262^e^
40%	14.2 ± 0.254^e^

Note: The lowercase superscript letter in the same column indicates a significant difference at 5% level.

### 3.2 Effect of *P. tolaasii* EPSs on physiological indices of *P. ostreatus*

Significant differences were observed in the proline and vitamin C contents of *P. ostreatus* at different EPS concentrations (Table 4). The contents increased with increasing EPS concentration: the lowest proline and vitamin C contents were recorded in the negative control (14.1 and 0.11 μg/g, respectively) and the highest were recorded at the EPS concentration of 40% (62.4 and 0.69 μg/g, respectively). Therefore, EPSs stimulate *P. ostreatus* to produce large amounts of proline and vitamin C.

**Table 4:** Effect of *P. tolaasii* EPSs on the intracellular physiological indices of *P. ostreatus*

EPS concentration (g/mL)	Proline content (μg/g) (±SD)	Vitamin C content (μg/g) (±SD)
0%	14.1 ± 0.152^d^	0.11 ± 0.011^g^
3%	15.3 ± 0.184^c,d^	0.25 ± 0.024^f^
5%	16.3 ± 0.193^c,d^	0.33 ± 0.014^e^
10%	17.6 ± 0.121^c,d^	0.40 ± 0.072^d^
20%	28.9 ± 0.661^c^	0.47 ± 0.053^c^
30%	39.6 ± 0.925^b^	0.59 ± 0.011^b^
40%	62.4 ± 0.514^a^	0.69 ± 0.041^a^

Note: The lowercase superscript letter in the same column indicates a significant difference at 5% level.

The activities of extracellular CMCase, α-amylase, and laccase and contents of proteins and sugars in *P. ostreatus* were significantly different at different ESP concentrations (Table 5). As the EPS concentration increased, the number of extracellular indicators decreased. The highest CMC enzyme activity was recorded in the negative control (54.6 U/L), whereas the lowest was recorded at an EPS concentration of 40% (20.4 U/L). The highest activity of α-amylase was recorded (37.8 U/L) in the negative control, and the lowest was recorded at an EPS concentration of 40% (18.9 U/L). The highest laccase activity was recorded in the negative control (74.7 U/L) and the lowest enzyme activity was at an EPS concentration of 40% (27.1 U/L). The highest protein content was 1.1 g/L in the negative control, and the lowest content was recorded at an EPS concentration of 40% (0.2 g/L). The lowest glucose utilisation rate in the medium was measured at an EPS concentration of 40% (0.6 g/L) and the highest in the negative control (23.7 g/L).

**Table 5:** Effect of *P. tolaasii* EPSs on physiological indices of *P. ostreatus*

EPS concentration (g/mL)	CMCase (U/L) (±SD)	α-Amylase (U/L) (±SD)	Laccase (U/L) (±SD)	Sugar utilisation rate (g/L) (±SD)	Protein content (gprot/L) (±SD)
0%	54.6 ± 0.011^a^	37.8 ± 0.630^a^	74.7 ± 0.001^a^	23.7 ± 0.101^a^	1.1 ± 0.100^a^
10%	41.3 ± 1.642^b^	29.9 ± 0.891^b^	53.8 ± 0.001^b^	20.9 ± 0.008^a^	0.6 ± 0.044^b^
30%	31.4 ± 1.051^c^	23.4 ± 0.678^c^	32.2 ± 0.003^c^	3.1 ± 0.058^b^	0.3 ± 0.037^c^
40%	20.4 ± 1.200^d^	18.9 ± 0.196^d^	27.1 ± 0.001^d^	0.6 ± 0.142^c^	0.2 ± 0.039^c^

Note: The lowercase superscript letter in the same column indicates a significant difference at 5% level. The sugar content in the control medium was 3.8 g/L.

## 4 Discussion

*P. tolaasii* is the cause of BBD in *A. bisporus* and *P. ostreatus* [25]. A toxin produced by *P. tolaasii* was identified as a lipodepsipeptide that forms an ion channel [26]. This toxin was named tolaasin, and seven of its analogues have been identified [25]. In addition to tolaasins, other toxic substances in *P. tolaasii* have been reported. In 1994, a compound from a *P. tolaasii* strain, characterised as an aminobenzene containing an amylamine group, was found to induce symptoms of the disease on the cap of *A. bisporus* [27]. Furthermore, in 1996, volatile organic compounds called tovsins were found in *P. tolaasii*; these compounds were found to be different from tolaasins and could induce changes in *P. ostreatus* and *F. velutipes* basidiomes [28]. The study in 2015 showed that *P. tolaasii* can produce volatile substances which induce *in vitro* mycelial growth inhibition of *P. ostreatus* and *P. eryngii*, and *A. bisporus* and *P. ostreatus* basidiome tissue blocked brown discoloration [16]. Therefore, in the present study, we extracted the polysaccharides from *P. tolaasii* and tested whether they affected the growth of *P. ostreatus* mycelia.

We found that the EPS of *P. tolaasii* caused strong physiological changes in *P. ostreatus* hyphae. Proline and vitamin C are important physiological indicators of an organism’s resistance to infection [29]. Proline is the most water-soluble amino acid and is highly hydrated. Its main function is to regulate the intracellular osmotic pressure by stabilising the cell membrane, which enhances an organism’s resistance. We showed that with the increase in the concentration of extracellular EPSs, the contents of proline and vitamin C increased significantly, indicating that the growth of *P. ostreatus* mycelia was suppressed by EPSs. Therefore, extensive regulation of proline and vitamin C contents is required to maintain the normal life activities of *P. ostreatus* mycelia. Moreover, with the increase in EPS concentration, the content of proline and vitamin C in *P. ostreatus* mycelia gradually increased, indicating that the conditions were becoming increasingly adverse. This finding was consistent with the physiological characteristics of plant stress resistance [29].

Proteins and carbohydrates are the major biological macromolecules responsible for various functions in organisms. CMCase can degrade cellulose to produce glucose and laccase can catalyse a variety of phenolic and non-phenolic compounds and simultaneously reduce oxygen to water [30]. α-Amylase (EC 3.2.1.1) catalyses the hydrolysis of α-1,4-glucosidic bonds in starch and related α-glucans [31]. In the present study, *P. tolaasii* EPS toxin caused a decrease in laccase, α-amylase, and cellulase activities of *P. ostreatus*, which also affected mycelial growth. The increase in EPS concentration decreased the protein content and enzyme activity, indicating that the structure and function of *P. ostreatus* mycelia were affected during growth, thereby inhibiting the normal growth of the hyphae.

The virulence factors of the lipopolysaccharides obtained from *Shigella* species include the endotoxic activities of the molecule’s lipid A component and the ability of the polysaccharide chain – the core and the O-antigenic polysaccharide – to provide the bacterium with resistance to host defence mechanisms, such as opsonisation, phagocytosis, and intracellular killing [32]. In mice, a *Yersinia pseudotuberculosis* O-polysaccharide-deficient derivative showed reduced virulence upon subcutaneous challenge [33]. The EPS of *P. tolaasii* caused strong physiological changes in *P. ostreatus* hyphae. EPS may contain lipopolysaccharides and other polysaccharides that affect the growth and physiology of *P. ostreatus* hyphae. However, the role of polysaccharides as virulence factors has not been elucidated and requires further investigations.

## 5 Conclusions

Compared to those in the control, the contents of vitamin C and proline at all EPS concentrations showed sustained increasing trends. The protein content and activities of CMCase, α-amylase, and laccase showed a downward trend with increasing EPS concentration. The EPS extracted from *P. tolaasii* not only reduced the growth rate, extracellular enzyme activity, and protein content of *P. ostreatus* but also increased its intracellular stress resistance to supplement the pathogenic mechanism of exocytosis in Bacteroides. We concluded that in addition to tolaasin, EPSs may also act as virulence factors in *P. tolaasii*.
